# 高原红细胞增多症诊断与治疗中国专家共识（2025年版）

**DOI:** 10.3760/cma.j.cn121090-20250326-00149

**Published:** 2025-07

**Authors:** 中华医学会血液学分会红细胞疾病学组 中国医院协会血液学机构分会

## Abstract

高原红细胞增多症（HAPC）指长期暴露于海拔2 500 m以上低氧环境引发的继发性红细胞过度增多症，以血红蛋白显著升高（男性≥210 g/L，女性≥190 g/L）为主要特征。HAPC临床表现为头痛、乏力、睡眠障碍等，易并发血栓及器官损伤。我国拥有广阔的高原面积，战略位置十分重要，移居和旅居高原人员日益增多。但既往缺乏这类疾病系统规范的诊疗指导，为进一步规范我国HAPC诊疗，中华医学会血液学分会红细胞疾病学组和中国医院协会血液学机构分会在广泛征求有关专家意见的基础上，结合最新循证证据及高原医学实践经验制定了本共识，明确分层诊疗策略有助于指导HAPC诊治：轻度采取低流量氧疗，中度采取药物和氧疗并重的综合治疗，重度推荐以红细胞单采术为主，辅以药物和氧疗。

## Abstract (en)

High-altitude polycythemia（HAPC）is defined as a secondary excessive erythrocytosis caused by prolonged exposure to hypoxic environments at altitudes above 2,500 meters, characterized primarily by significantly elevated hemoglobin levels（≥210 g/L in males and ≥190 g/L in females）. Clinically, HAPC manifests as headache, fatigue, sleep disturbances, and is prone to complications such as thrombosis and organ damages. China encompasses extensive high-altitude regions of critical strategic significance, with a growing population of residents and travelers relocating or temporarily staying in these areas. However, there has been a lack of systematic and standardized clinical guidelines for the diagnosis and management of high-altitude-related diseases. To standardize the diagnosis and treatment of HAPC in China, the Red Blood Cell Disease Group, Chinese Society of Hematology, Chinese Medical Association and Chinese Hospital Association Hematology Branch formulated this consensus based on extensive expert consultation, integrating the latest evidence-based findings and practical experience in high-altitude medicine. The consensus defines a stratified diagnostic and therapeutic strategy to guide the clinical management of HAPC: mild cases are managed with low-flow oxygen therapy; moderate cases require combined pharmacological and oxygen therapy; and severe cases are recommended for erythrocytapheresis as the primary intervention, supplemented by pharmacotherapy and oxygen support.

医学高原一般指海拔2 500 m及以上的高原或高山地区，全世界约1.4亿人长期生活在上述地区[1]。中国拥有广阔的高原地区，其中青藏高原面积约为250万平方公里，平均海拔为4 000～5 000 m，被誉为“世界屋脊”和“第三极”。我国常年在海拔2 500 m以上高原居住的人口为6 000～8 000万人[2]。高原自然环境恶劣，为适应低压低氧的环境，机体红细胞反应性增多，但如果过度反应则演变为高原红细胞增多症（high altitude polycythemia, HAPC）。HAPC通常指居住在高原6个月以上，因缺氧导致的以红细胞过度增多为主要特征，进而出现以头痛、头昏、乏力、心悸、睡眠障碍为主要症状的临床综合征[1]，是慢性高原病的一种类型。需要说明的是，HAPC的高原海拔一般认为是2 500 m以上，但是也有不同描述，主要应关注高原缺氧导致红细胞过度增多，血液黏滞度异常增高，微循环障碍，组织严重缺氧，易导致血栓形成或局部组织坏死等并发症。

高原移居人群是HAPC的高发人群，显著高于世居藏族和其他民族人群。在青藏高原海拔3 000 m地区，HAPC发病率可高达24.0％，且随海拔的升高而增加[3]–[5]，在海拔超过5 000 m的地区，HAPC发生率可高达63.3％[6]。发病的影响因素主要包括海拔高度、遗传因素、性别、年龄、职业、高原居住时间、生活习惯、感染与炎症、身体质量指数、运动、营养和微量元素等[7]。HAPC发病的核心是环境低氧，但缺氧后人体各系统对环境低氧应答的发病机制尚不完全清楚，一般认为与基因、RNA、蛋白表达谱及炎症因素等有关[8]–[12]。HAPC是引发其他慢性高原病的基础，严重影响高原人群健康，但目前尚缺乏规范而系统的临床诊治的专家共识，近5年国内外也仅有HAPC的中医临床诊疗指南[13]及2023年美国梅奥诊所发表的非JAK2突变的红细胞增多症的诊治指南[14]。因此，随着对HAPC认识的提高，为了更好地规范中国HAPC的诊治，特撰写HAPC诊断与治疗中国专家共识。

一、临床表现

1. 症状：HAPC是一组由缺氧和血液淤滞引起的临床综合征，临床表现无特异性，涉及多系统多器官的症状，包括疲劳、全身乏力、头痛、头昏、心悸、气喘、胸痛、视觉改变、健忘、肌肉关节疼痛、睡眠障碍、女性月经不调和性功能异常等，常伴有高血压。脱离低压低氧环境后，症状可逐渐消失，但重返高原后又可能复发。

2. 体征：HAPC患者的典型体征为紫绀，特有的“高原多血面容”表现为面部毛细血管扩张成紫红色条纹。紫绀在口唇、指甲等处常见。可伴杵状指、肝脾肿大、下肢水肿、结膜充血等。

3. 相关辅助检查：

（1）血常规：血红蛋白（HGB）浓度升高是本病最突出的特征，也是重要的危险度分层指标，通常作为选择治疗方案的依据。HAPC患者HGB浓度、血细胞比容、红细胞计数均增高，但白细胞计数和血小板计数通常在正常范围内，血小板计数一般低于同海拔健康人群[15]–[17]。

（2）骨髓细胞学检查：HAPC患者的骨髓细胞学表现为正常骨髓象或红系增生明显活跃，幼红细胞比例增高，粒系比例相对减少。

（3）血液生化检查：HAPC患者的促红细胞生成素（EPO）水平较平原地区健康人群明显升高，与高原地区健康人群相当或轻度升高[18]–[21]。

（4）心电图和胸部X线片：单纯HAPC一般不引起心电图和X线片改变。当合并肺动脉高压时，可出现心电轴右偏及右心室肥厚，X线片可表现为右心室增大、肺动脉段凸出和右下肺动脉增宽[22]。

（5）血气分析：与同海拔健康人群相比，HAPC患者血气分析表现为低氧血症和相对性高碳酸血症。患者pH、动脉血氧分压（PaO_2_）和动脉血氧饱和度（SaO_2_）均较低，虽然动脉血二氧化碳分压（PaCO_2_）在正常范围内，但仍较同海拔健康人群高[23]。低通气导致的相对性高碳酸血症可能是HAPC的病因之一[24]。

（6）肾功能检查：HAPC患者血尿酸、尿微量白蛋白较同海拔健康人群高，而肾小球滤过率无显著差异。肾血流量较低和长期高滤过分数可能导致慢性肾脏损害[25]。

二、诊断与鉴别诊断

1. 诊断：推荐使用2023年3月1日实施的中华人民共和国国家军用标准GJB 10684-2022《慢性高原病的诊断与处理原则》并参照2004年第六届国际高原医学会议标准中HAPC的诊断标准[26]–[28]，即符合以下条件：

（1）居住在海拔2 500 m以上的时间>6个月；

（2）女性标准为HGB≥190 g/L，男性标准为HGB≥210 g/L；

（3）有头痛、心悸、气短、睡眠障碍等症状；

（4）排除其他类型红细胞增多症。

除以上诊断标准外，如果患者返回平原后症状减轻，病情逐渐好转，再次进入高原后病情复发，可支持HAPC的诊断。如果无相关症状但符合其他诊断标准，或有相关症状但HGB未达到诊断标准，均不建议诊断为HAPC。

2. 鉴别诊断：HAPC是继发性红细胞增多症的一种，排除其他可引起红细胞增多的疾病是诊断HAPC的前提条件。首先HAPC应与真性红细胞增多症相鉴别，HAPC患者的骨髓细胞学可表现为正常骨髓象或红系增生明显活跃；而真性红细胞增多症的骨髓活检表现为与年龄不符的红细胞过多伴三系增生，EPO显著下降，基因突变检测最常发现JAK2 V617F或JAK2第12号外显子基因突变，CARL或MPL基因突变少见[29]。此外还需与其他继发性红细胞增多症鉴别，如慢性肺部疾病（肺气肿、慢性支气管炎、支气管扩张、囊性纤维化、癌症等）、先天性心脏病（右向左分流型）、阻塞性睡眠呼吸暂停低通气综合征、肾动脉硬化或狭窄、多囊肾及肾移植后、其他加重低氧血症的潜在慢性疾病、少见的遗传相关氧敏感通路突变基因［EpoR、VHL、PHD2、HIF-2α、HBA1、HBA2和（或）BPGM］导致的红细胞增多症[30]。

3. 危险度分层：推荐使用2004年第六届国际高原医学会议上发布的青海计分系统（表1），目前依然被广泛应用于HAPC患者的危险度分层[26],[31]。青海计分系统纳入气喘或心悸、失眠、紫绀、血管扩张、感觉异常、头痛、耳鸣及HGB共8个指标进行计分。其中，≤10分为轻度HAPC，11～14分为中度HAPC，≥15分为重度HAPC。临床应用时建议根据危险度分层结果选择不同的治疗策略。

**表1:** 高原红细胞增多症（HAPC）青海计分系统

症状或体征	严重程度	计分
气喘/心悸	无气喘/心悸	0
	轻度气喘/心悸	1
	中度气喘/心悸	2
	重度气喘/心悸	3
失眠	睡眠正常	0
	不能正常入眠	1
	睡眠不足，时睡时醒	2
	无法入眠	3
紫绀	无紫绀	0
	轻度紫绀	1
	中度紫绀	2
	重度紫绀	3
静脉扩张	无静脉扩张	0
	轻度静脉扩张	1
	中度静脉扩张	2
	重度静脉扩张	3
感觉异常	无感觉异常	0
	轻度感觉异常	1
	中度感觉异常	2
	重度感觉异常	3
头痛	无头痛	0
	轻度头痛	1
	中度头痛	2
	重度头痛	3
耳鸣	无	0
	轻度	1
	中度	2
	重度	3
HGB	男性：>180 g/L且<210 g/L	0
	女性：>160 g/L且<190 g/L	0
	男性：≥210 g/L	3
	女性：≥190 g/L	3

**注** 轻度HAPC：计分≤10分；中度HAPC：计分≥11分且≤14分；重度HAPC：计分≥15分

三、治疗

治疗原则：HAPC的病因是环境低氧低压，因此最有效的治疗建议首选转至低海拔或平原地区，尤其对于重度HAPC患者应尽快脱离低氧环境。而部分患者由于个人和工作等原因不能或不想下降海拔，本文主要就返回平原地区前或无法脱离高原环境的患者提出治疗策略。建议轻度HAPC患者采取低流量吸氧治疗，定期复查动态观察。对于中度HAPC患者，采取药物和氧疗并重的综合治疗策略。对于HGB>250 g/L、重度、复发难治性或凝血功能异常的HAPC，采取以红细胞单采为主，辅以药物和氧疗的治疗策略。HAPC的诊疗路径见图1。

**图1:**
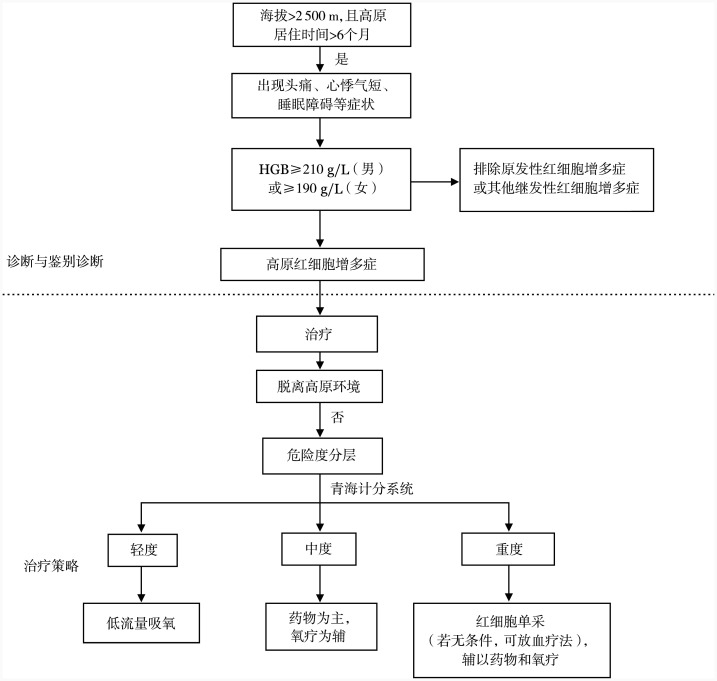
高原红细胞增多症诊疗路径图

（一）氧疗

推荐吸氧是HAPC的基础治疗，可明显减轻HAPC患者的症状。轻中度HAPC患者可选择低流量吸氧；重度、迟发型、复发难治性HAPC患者优先应用高压氧治疗[32]–[33]，合并高压氧禁忌证时可选择低流量吸氧。有条件者可选择持续富氧房间睡眠。

1. 低流量吸氧：可使用面罩吸氧或鼻导管吸氧，流量1～2 L/min，1～2 h/次，2次/d。也可采用如下方案：1～2 L/min，1 h/次，3～4次/d[34]。单纯吸氧仅对轻症患者有效。对于中重度HAPC，建议联合药物或红细胞单采等治疗。

2. 高压氧疗：建议高压氧疗与药物治疗相结合，一般2～4个疗程后可使患者的HGB降低20～40 g/L，每2个疗程间隔1周。各氧疗方案具体参数为：（1）治疗压力250 kPa，面罩给纯氧，稳压时间20 min×2，间歇10 min，1次/d，10次为1个疗程[35]。（2）治疗压力200 kPa，面罩给纯氧，稳压时间40 min×2，间歇10 min，1次/d，10～15次为1个疗程[36]–[37]。

高压氧疗的绝对禁忌证包括：（1）未处理的气胸、纵隔气肿；（2）活动性内出血及出血性疾病；（3）肺大疱；（4）结核性空洞并咯血；（5）同时服用双硫仑[33]。相对禁忌证包括：（1）胸部外科手术围手术期；（2）呼吸道传染性病毒感染；（3）中耳手术围手术期；（4）未控制的癫痫；（5）高热；（6）先天球形红细胞增多症；（7）幽闭恐惧症；（8）颅底骨折伴脑脊液漏；（9）妊娠3个月以内不建议多次高压氧治疗；（10）未控制的高血压；（11）血糖控制不稳定的糖尿病；（12）视网膜剥离或青光眼（闭角型）；（13）心脏Ⅱ度以上房室传导阻滞；（14）心动过缓（小于50次/min）；（15）支气管扩张症；（16）严重肺气肿；（17）未经处理的恶性肿瘤；（18）同时服用抗肿瘤药物[33]。

（二）药物治疗

1. 西药

（1）抑制红细胞生成的药物

雌激素及其类似物：雌激素有抑制红细胞生成的作用[38]，具体用法：己烯雌酚1 mg/次，2次/d，口服，4周为1个疗程[39]。但由于雌激素有女性化不良反应，男性患者常难以接受而较少使用。此外，长期应用雌激素有深静脉血栓风险，需密切关注。建议使用大豆异黄酮替代雌激素治疗，大豆异黄酮为大豆提取物，有雌激素样活性、抑制血栓形成、抗氧化、抗癌、抑制血管平滑肌收缩和降血压等多种生物活性，具体用法：大豆异黄酮胶囊20 mg/次，2次/d，口服，3个月为1个疗程[34],[40]–[41]。

碳酸酐酶抑制剂：乙酰唑胺可降低血清EPO和血细胞比容，改善对缺氧的通气反应。具体用法为250 mg，口服，1次/d，21 d为1个疗程[21],[42]–[44]。相较于乙酰唑胺，同类药物醋甲唑胺的疲劳不良反应更小[45]。

（2）抗凝及活血化瘀药物：HAPC患者红细胞失代偿性增高导致血液淤滞，易产生血栓[46]–[47]。可酌情服用阿司匹林肠溶片（75～100 mg/d）抗血小板凝聚，防止血栓形成和栓塞。一旦出现血栓，可使用肝素抗凝[48]。目前认为肝素钠联合藻酸双酯钠（PSS）的疗效优于单药[49]。推荐用量为肝素钠12 500 U，1次/d，静脉泵入；PSS 200 mg，1次/d，静脉滴注。PSS的常见不良反应有呕吐、头晕等，不耐受者可将PSS剂量减低至100 mg。肝素半衰期短，应用不方便，推荐使用低分子肝素。低分子肝素钠4 000～6 000 U，2次/d，皮下注射。

（3）其他机制药物：其他药物如血管紧张素转化酶抑制剂（ACEI）类药物、甲基黄嘌呤、肾上腺素受体阻滞剂和多巴胺受体拮抗剂、中枢或外周呼吸兴奋剂等在其他红细胞增多症中有效[50]–[51]，但在HAPC中临床应用较少，仅可考虑作为备选药物。

2. 中医药和藏药：建议中医药与其他治疗方式联合应用，发挥免疫调节、活血化瘀的作用。常用的复方中药制剂有：复方丹参片（滴丸）、心脑欣胶囊、三七制剂等[52]。推荐用量为：（1）利舒康胶囊：1 g/次，口服，3次/d；（2）复方丹参片：3片/次，口服，3次/d；（3）丹参滴丸：10丸/次，口服或舌下含服，3次/d；（4）心脑欣胶囊：2粒/次，2次/d，口服。（5）血塞通片：1～2片/次，口服，3次/d。

目前多种临床有效的藏药成分从高原耐缺氧植物中提取，因此藏医药在HAPC的治疗中具有独特的优势[53]。常用的复方藏药有多血康胶囊、二十五味余甘子丸、三果汤、左木阿汤等，单方藏药有红景天胶囊、沙棘等，可选用1～2种治疗。推荐用法为：（1）多血康胶囊4粒/次，口服，3次/d，1个月为1个疗程；（2）红景天胶囊2粒/次，口服，3次/d，1个月为1个疗程，共3个疗程，2个疗程之间间隔1个月。

（三）红细胞单采术及放血疗法

1. 红细胞单采：建议HAPC患者HGB>250 g/L时启动红细胞单采治疗[54]，若临床症状明显或出现凝血功能异常等不良因素，单采治疗的指征可放宽至HGB≥210 g/L[55]。与传统的放血疗法相比，红细胞单采术具有多种优势，其1年有效率高达88.5％，缓解时间较长，复发率较低[56]–[59]。因此优先推荐采用红细胞单采术，若缺乏红细胞单采条件，可选择放血疗法。

红细胞单采术的具体操作流程是：（1）治疗前静推10％葡萄糖酸钙溶液10 ml预防低钙血症。（2）在单采仪器中输入患者性别、身高、体重、外周血血细胞比容等参数，计算预定全血循环体积。连续循环流速维持在20～40 ml/min，3个循环，循环血量1 300～1 500 ml，一个循环采集红细胞200 ml，离心参数5 200 r/min，1次采集量为500～600 ml红细胞（抗凝剂为复方枸橼酸钠注射液，抗凝剂与全血比例为1∶11～1∶13）。（3）采集时补充等量的生理盐水（或复方氯化钠注射液），同时补充10％葡萄糖酸钙注射液10～20 ml，操作中应注意枸橼酸盐中毒等不良反应的观察和处理。若发生严重麻木、抽搐等症状，应停机至少15 min，待患者症状好转后继续。对于枸橼酸盐敏感的患者，可改用肝素抗凝[60]。

2. 放血疗法：放血疗法近几年逐步被红细胞单采术取代，但鉴于其价格优势，且技术要求低，放血疗法仍是无红细胞单采术条件患者的第一选择。目前认为同时具备以下3个指征时可采用放血疗法：HGB>250 g/L；血细胞比容>70％；有血管栓塞或脑缺血先兆[61]。若临床症状明显，HGB标准可适当放宽，并酌情调整放血容量。具体方法：每次放血300～400 ml，建议放血时间间隔3个月，也可根据实际情况调整。放血疗法仅在短期内改善症状，可能加重肺动脉高压，因此仅限于重症患者。临床中普遍观察到放血治疗后患者返回高海拔地区症状会再次出现，血细胞比容等指标通常会在数周内达到并超过治疗前水平。

四、疗效评估

HAPC的疗效评估尚无统一标准，建议在临床初步评估时采用如下疗效标准[62]：显效：临床症状、体征显著改善，实验室检查恢复正常；好转：临床症状、体征减轻，实验室检查显著好转；无效：临床症状、体征、实验室检查等无改变或恶化。其中，实验室检查包括红细胞计数、血细胞比容和HGB浓度等。需要指出的是，上述疗效标准未定义“显著”，且临床症状较主观，安慰剂对青海计分系统评分的影响较大[21],[44]。随着技术的进步，新的实验室检查手段已运用于临床，故本共识推荐在精准评估疗效时采用综合疗效评判标准（表2）。

**表2:** 高原红细胞增多症（HAPC）综合疗效评判标准

疗效标准	定义
完全缓解（CR）	需同时满足： ①血常规：HGB<210 g/L（男）或<190 g/L（女），HCT恢复至同海拔健康人群水平（HCT<55.0％）[15],[23],[63]； ②症状及体征消失； ③血气分析：PO_2_、SaO_2_及PCO_2_达到同海拔健康人群水平（PO_2_>50 mmHg，SaO_2_>85.0％，PCO_2_<35.0 mmHg[23]）； ④无疾病进展，无出血或血栓事件
部分缓解（PR）	需同时满足： ①血常规：HGB较前下降（至少10％），但仍≥210 g/L（男）或≥190 g/L（女）；或HCT降低至少10％； ②青海计分系统评分较前下降，症状及体征较前减轻； ③血气分析：PO_2_、SaO_2_较前增加，PCO_2_较前降低，但未达到同海拔健康人群水平； ④无疾病进展，无出血或血栓事件
未缓解（NR）	疗效未达到PR
疾病进展（PD）	①HGB、HCT较前增加； ②单纯HAPC出现肺动脉高压或高原性心脏病

**注** HCT：血细胞比容；PO_2_：血氧分压；SaO_2_：血氧饱和度；PCO_2_：血二氧化碳分压

五、预防

1. 生活方式改善：保持稳定的情绪，保证充足的睡眠，辅以规律轻中度有氧运动[1],[64]–[65]。调整饮食结构，目前普遍推荐多食蔬菜水果，低脂少盐饮食。推荐饮用藏族饮食酥油茶[66]。筛查并去除高危因素，积极治疗基础疾病。戒烟戒酒，积极减重。

2. 居家氧疗：推荐自备小型制氧机居家氧疗。建议间断低流量吸氧，流量1～2 L/min，1 h/次，3～4次/d[67]。或持续富氧房间睡眠。

3. 呼吸训练：缓慢深呼吸，6～8次/min，连续3～5 min。
